# Zinc Attenuates the Cytotoxicity of Some Stimuli by Reducing Endoplasmic Reticulum Stress in Hepatocytes

**DOI:** 10.3390/ijms20092192

**Published:** 2019-05-03

**Authors:** Masashi Kusanaga, Shinji Oe, Noriyoshi Ogino, Sota Minami, Koichiro Miyagawa, Yuichi Honma, Masaru Harada

**Affiliations:** Third Department of Internal Medicine, School of Medicine, University of Occupational and Environmental Health, Kitakyushu 807-8555, Japan; ooes@med.uoeh-u.ac.jp (S.O.); n-ogino@med.uoeh-u.ac.jp (N.O.); s-minami@med.uoeh-u.ac.jp (S.M.); koichiro@med.uoeh-u.ac.jp (K.M.); y-homma@med.uoeh-u.ac.jp (Y.H.)

**Keywords:** autophagy, copper, endoplasmic reticulum stress, fatty acid, Mallory–Denk body, Wilson disease, zinc acetate

## Abstract

Zinc is an essential trace element and plays critical roles in cellular integrity and biological functions. Excess copper induced both oxidative stress and endoplasmic reticulum (ER) stress in liver-derived cultured cells. Excess copper also induced impairment of autophagic flux at the step of autophagosome–lysosome fusion, as well as Mallory–Denk body (MDB)-like inclusion body formation. Zinc ameliorated excess copper-induced impairment of autophagic flux and MDB-like inclusion body formation via the maintenance of ER homeostasis. Furthermore, zinc also ameliorated free fatty acid-induced impairment of autophagic flux. These results indicate that zinc may be able to protect hepatocytes from various ER stress-related conditions.

## 1. Introduction

Zinc is an essential trace element and plays critical roles in cellular integrity and various biological functions. The majority of zinc is located in the skeletal muscle and bone, and the rest is distributed in widespread tissues such as the liver, prostate gland, and skin [1]. Zinc itself has no redox capacity and acts as a cofactor for many enzymes and proteins involved in anti-oxidant, anti-inflammatory, and anti-apoptotic functions [2]. Conditioned zinc deficiency is present in many chronic diseases such as rheumatoid arthritis, cancers, and liver diseases, which are mainly associated with chronic inflammation and oxidative stress [3,4]. Some studies suggested that many liver diseases, especially alcoholic liver disease and viral hepatitis, are associated with hypozincemia or zinc deficiency [5,6,7]. In addition, recent studies demonstrated that zinc supplementation partly improved liver injury and provided anti-fibrotic effects, and subsequently reduced the incidence of hepatocellular carcinoma [8,9,10]. Zinc acetate has been commonly used to treat patients with Wilson disease because it inhibits absorption of copper from the intestinal epithelia [11]. We have previously reported that excess copper induced endoplasmic reticulum (ER) stress as well as oxidative stress, contributing to disease progression in Wilson disease [12]. Excess copper was found to induce the accumulation of unfolded or misfolded proteins within the ER lumen. Moreover, we have confirmed that zinc acetate dampened ER stress and hepatotoxicity, including DNA damage and apoptosis induced by excess copper. Thus, we speculate that zinc acetate may play an important role in the protection of ER homeostasis in conditions of other liver diseases.

Macroautophagy (hereafter referred to as autophagy) plays a crucial role for intracellular quality control of proteins and various organelles in various tissues [13]. Impairment of autophagy results in accumulation of abnormal proteins and damaged organelles, and induces the formation of intracellular inclusions, which has been implicated in a number of human diseases [14,15]. Upon induction of autophagy, an isolation membrane called the “phagophore” elongates and encloses a part of the cytoplasm and/or damaged organelles, which results in the formation of an autophagosome. The autophagosome then fuses with a lysosome to form an autolysosome, and the contents are degraded [16]. As described above, excess copper exposure induces oxidative stress and ER stress. Oxidative stress is a critical mediator of ER dysfunctions [17]. Hepatic cells cope with ER stress by an adaptive or protective response, termed the “unfolded protein response” (UPR). If activation of the UPR is insufficient, the ER stress leads to pathological processes such as hepatic lipid accumulation, inflammation, and cell death, all of which can cause various liver diseases [18]. In this situation, the demand on protein synthesis and degradation machinery, including autophagy, increases to maintain cellular homeostasis. Autophagy and ER stress are interconnected, and recent reports have revealed that ER stress can either induce or inhibit autophagy [19,20]. We have previously reported that proteasome dysfunction-induced ER stress activated autophagy to eliminate aberrant proteins, whereas free fatty acids (FFAs)-induced ER stress impaired autophagy [15,21]. These findings led us to further evaluate the link between excess copper load and alterations in autophagy.

In this study, we evaluate whether zinc acetate can play a beneficial role in some situations, including copper overload, proteasome dysfunction, and lipid overload, with a particular emphasis on ER stress and autophagy in cultured hepatocytes.

## 2. Results

### 2.1. Zinc Acetate Reduces Pharmacological Endoplasmic Reticulum Stress

**Figure 1:**
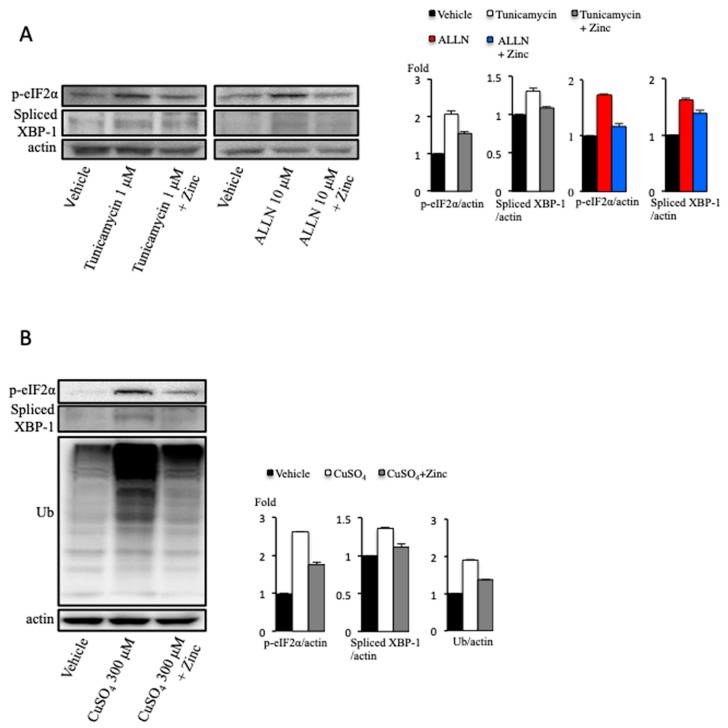
Effect of copper on endoplasmic reticulum (ER) stress. (**A**) Huh7 cells were treated with tunicamycin 1 μM or acetyl-leucyl-leucyl-norleucinal (ALLN) 10 μM for 12 h, and zinc acetate (200 μM) was added 2 h before these treatments. Densitometry analysis of indicated proteins is shown on the right. (**B**) OUMS-29 cells were treated with copper at the concentration of 300 μM for 12 h, and zinc acetate (200 μM) was added 2 h before copper sulfate treatment. Densitometry analysis of indicated proteins is shown on the right.

We initially examined the effect of zinc acetate on pharmacological ER stress in cultured hepatocytes. ER stress was evaluated by detection of the ER stress surrogate markers, spliced X-box binding protein 1 (XBP1) and the phosphor-α-subunit of eukaryotic initiation factor 2α (p-eIF2α). Pharmacological ER stress was induced by tunicamycin and acetyl-leucyl-leucyl-norleucinal (ALLN). Tunicamycin blocks the initial step of glycoprotein biosynthesis in the ER through the inhibition of the UDP-*N*-acetylgucosamine-dolichol phosphate *N*-acetylglucosamine-1-phosphate transferase [22]. ALLN, one of the proteasome inhibitors (PIs), also induces ER stress by the accumulation of misfolded proteins within the ER. Both spliced XBP1 and p-eIF2α expression were increased in cultured hepatocytes treated with tunicamycin for 12 h at the concentration of 1 μM as well as ALLN for 12 h at 10 μM (Figure 1A). The elevations of spliced XBP1 and p-eIF2α expression induced by tunicamycin and ALLN were both suppressed by zinc acetate treatment for 2 h at the concentration of 200 μM (Figure 1A). Similar results were found in excess copper-treated cultured hepatocytes, treated for 12 h at the concentration of 300 μM (Figure 1B). These results indicated that zinc acetate attenuated not only excess copper-induced ER stress but also pharmacological ER stress. Next, to clarify whether the ameliorative effect of zinc acetate on excess copper-induced ER stress was accompanied by a reduction of abnormal proteins, we monitored the levels of ubiquitinated proteins by immunoblotting analysis. Ubiquitinated proteins, known as substrates for the ubiquitin–proteasome system, are associated with several types of stress, including ER stress. We found that the level of ubiquitinated proteins increased in the excess copper-treated cultured hepatocytes, and that this effect was ameliorated in the presence of zinc acetate (Figure 1B).

### 2.2. Excess Copper Impairs Autophagic Flux at the Step of Autophagosome–Lysosome Fusion

**Figure 2:**
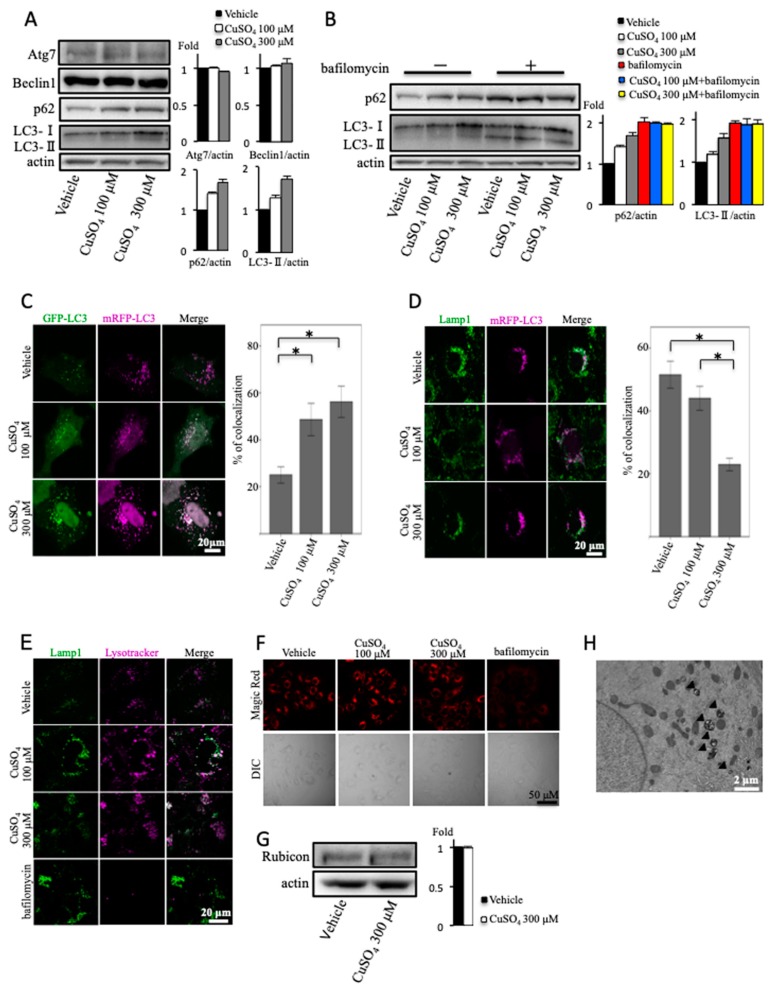
Effect of copper on autophagy. (**A**) OUMS-29 cells were treated with copper at concentrations of 100 or 300 μM for 12 h. Densitometry analysis of indicated proteins is shown on the right. (**B**) Light chain 3 (LC3) turnover assay. OUMS-29 cells were treated with copper at concentrations of 100 or 300 μM for 12 h with or without 50 nM of bafilomycin A1. Densitometry analysis of indicated proteins is shown on the right. (**C**) OUMS-29 cells were transfected with monomeric red fluorescent protein (mRFP)–green fluorescent protein (GFP) tandem fluorescent-tagged LC3 plasmids, and were treated with copper at concentrations of 100 or 300 μM for 12 h. Colocalizations of mRFP and GFP signals were measured by counting an overall total of 30 to 40 cells over the course of three independent experiments. Colocalization was shown as the percentage of merged signals in the total number of mRFP signals. Data are expressed as means ± SEM. (**D**) OUMS-29 cells were transfected with mRFP-LC3, and were treated with copper at concentrations of 100 or 300 μM for 12 h. Then, immunofluorescence staining for lysosomal-associated membrane protein 1 (Lamp1) was performed. (**E**) Effect of copper on lysosomal function. OUMS-29 cells, treated with copper at concentrations of 100 or 300 μM with or without bafilomycin A1 (50 nM) for 12 h, were loaded with 1 μM lysotracker Red for 1 h, followed by fixation, and then immunofluorescence staining for Lamp1 was performed. (**F**) Effect of copper on lysosomal function. OUMS-29 cells, treated with copper at concentrations of 100 or 300 μM with or without bafilomycin A1 (50 nM) for 12 h, were loaded with Magic Red Cathepsin B reagent for 1 h, and then analyzed by microscopy. (**G**) Huh7 cells were treated with copper at the concentration of 300 μM for 12 h. Densitometry analysis of indicated proteins is shown on the right. (**H**) Transmission electron micrograph of a Wilson disease patient. Arrowheads indicate the autophagosomes.

We next examined the effect of excess copper on autophagy in cultured hepatocytes. First, we analyzed the autophagy in cultured hepatocytes treated with excess copper by immunoblotting of microtubule-associated protein light chain 3 (LC3)-II and SQSTM1/p62 protein. The amount of LC3-II is clearly associated with the amount of autophagosomes and is widely used to monitor autophagy. p62 is an autophagy substrate. An increase in LC3-II level was observed in excess copper-treated cells, coincident with an increase of p62 (Figure 2A). In addition, the expression of Beclin1 and autophagy-related gene 7 (Atg7) proteins, required for the formation of autophagosomes [23,24], was unaltered between vehicle and excess copper (Figure 2A). The increase of LC3-II level is not always indicative of autophagy induction, because either increased generation of autophagosomes or a blockage in lysosomal degradation can induce this phenomenon [25]. In most cases, however, the accumulation of p62 indicates a decrease in autophagic degradation. Next, we analyzed autophagic flux by LC3 turnover assay, one of the principal methods to evaluate autophagic flux. In the presence of bafilomycin A1, a vacuolar H^+^-ATPase inhibitor, autophagic degradation is completely blocked, resulting in the accumulation of LC3-II and p62 [26]. Accordingly, the differences in the amount of LC3-II in the presence or absence of lysosomal inhibitors represent the amount of LC3-II that is delivered to lysosomes for degradation [27]. In the present study, concomitant treatment with bafilomycin A1 and excess copper increased LC3-II and p62 levels, and these expression levels were similar compared with bafilomycin A1 alone (Figure 2B). This meant that LC3-II accumulation upon excess copper treatment was due to impairment of autophagic flux at the step of autophagosome–lysosome fusion or lysosomal degradation, but not due to impaired formation of autophagosomes. To confirm this hypothesis, monomeric red fluorescent protein (mRFP)–green fluorescent protein (GFP)-LC3 transfection was performed. As a result of the different pH stabilities of the green and red fluorescent proteins, the GFP-LC3 loses its fluorescent signal within the acidic lysosomal environment, but the mRFP-LC3 signal persists [28]. In the cells transfected with mRFP–GFP-LC3, colocalization of GFP-LC3 with mRFP-LC3, observed as white signals, indicates autophagosomes, whereas only-mRFP-LC3 puncta indicate autolysosomes. As shown in Figure 2C, the colocalization of mRFP-LC3 and GFP-LC3 was significantly increased in the excess copper-treated cells. This result also indicated that excess copper impaired autophagosome–lysosome fusion or lysosomal dysfunction.

Lysosomal-associated membrane protein 1 (Lamp1) localizes in the late endosome and lysosome [29]. We analyzed the colocalization of mRFP-LC3 with Lamp1 to evaluate autophagosome–lysosome fusion (autolysosome formation). Compared with vehicle, excess copper significantly decreased the colocalization of mRFP-LC3 with Lamp1, suggesting that excess copper impaired autophagic flux at the step of autophagosome–lysosome fusion (Figure 2D).

Subsequently, we examined the effect of excess copper on lysosomal functions. First, we used Lysotracker Red, which is a fluorescent dye accumulating in acidic components [30]. We compared the locations of Lamp1 and Lysotracker Red. We found the colocalization of Lamp1 with Lysotracker Red in excess copper-treated cells but not in bafilomycin A1-treated cells (Figure 2E). These results indicated that lysosomal acidification was not impaired by excess copper treatment. Next, we used a Magic Red cathepsin B detection kit to evaluate the cathepsin B enzyme activity. The red fluorescence intensity in excess copper-treated cells did not differ from vehicle, whereas bafilomycin A1 reduced the fluorescence intensity (Figure 2F). These results suggested that neither lysosomal acidification nor protease activity was impaired in excess copper-treated cells.

Rubicon (Run domain Beclin-1 interacting and cysteine-rich containing protein) was identified as a component of the Class III phosphatidylinositol-3 kinase (PI3K) complex, and as a negative regulator of canonical autophagy and endosomal trafficking. In 2009, two research groups simultaneously identified KIA0226 as a novel Beclin1-binding protein, now known as Rubicon [31,32]. The expression of Rubicon protein was unaltered by the treatment with excess copper (Figure 2G). These results indicated that excess copper impaired autophagic flux in a Rubicon-independent manner.

The expression of autophagosomes was increased in hepatocytes from a patient with Wilson disease when observed by transmission electron microscopy (Figure 2H).

### 2.3. Zinc Acetate and 4-Phenylbutyrate Ameliorate Excess Copper-Induced Endoplasmic Reticulum Stress and Impairment of Autophagic Flux

**Figure 3:**
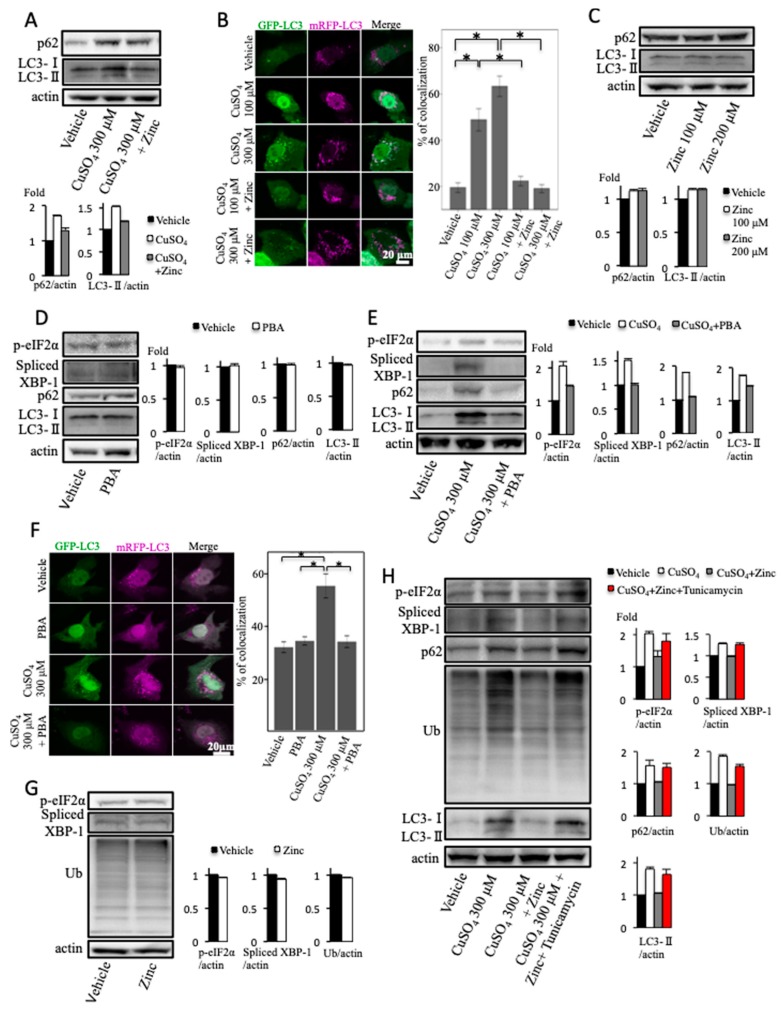
Effect of copper and zinc acetate treatment on autophagy. (**A**) Huh7 cells were treated with copper at the concentration of 300 μM for 12 h, and zinc acetate (200 μM) was added 2 h before copper sulfate treatment. Densitometry analysis of indicated proteins is shown on the bottom. (**B**) OUMS-29 cells were transfected with monomeric red fluorescent protein (mRFP)–green fluorescent protein (GFP) tandem fluorescent-tagged light chain 3 (LC3) plasmids. They were treated with copper at concentrations of 100 or 300 μM for 12 h, and zinc acetate (200 μM) was added 2 h before copper sulfate treatment. Colocalizations of mRFP and GFP signals were measured by counting an overall total of 30 to 40 cells over the course of three independent experiments. Colocalization was shown as the percentage of merged signals in the total number of mRFP signals. Data are expressed as means ± SEM. (**C**) Huh7 cells were treated with zinc acetate at concentrations of 100 or 200 μM for 2 h. Densitometry analysis of indicated proteins is shown on the bottom. (**D**) Huh7 cells were treated with 4-phenylbutyrate (PBA) at the concentration of 5 mM for 2 h. Densitometry analysis of indicated proteins is shown on the right. (**E**) Huh7 cells were treated with copper at the concentration of 300 μM for 12 h, and PBA (5 mM) was added 2 h before copper sulfate treatment. Densitometry analysis of indicated proteins is shown on the right. (**F**) OUMS-29 cells were transfected with mRFP–GFP tandem fluorescent-tagged LC3 plasmids. They were treated with copper at the concentration of 300 μM for 12 h, and PBA (5 mM) was added 2 h before copper sulfate treatment. Colocalizations of mRFP and GFP signals were measured by counting an overall total of 30 to 40 cells over the course of three independent experiments. Colocalization was shown as the percentage of merged signals in the total number of mRFP signals. Data are expressed as means ± SEM. (**G**) Huh7 cells were treated with zinc acetate at the concentration of 200 μM for 2 h. Densitometry analysis of indicated proteins is shown on the right. (**H**) Huh7 cells were treated with copper at the concentration of 300 μM and tunicamycin at the concentration of 0.5 μM for 12 h, and zinc acetate (200 μM) was added 2 h before copper sulfate treatment. Densitometry analysis of indicated proteins is shown on the right.

We investigated the effects of zinc acetate on the impairment of autophagic flux induced by copper treatment. Zinc acetate reduced the excess copper-induced elevation of LC3-II and p62 protein (Figure 3A). In the mRFP–GFP-LC3 transfected cells, zinc acetate significantly reduced the colocalization efficiency of mRFP-LC3 with GFP-LC3 induced by excess copper treatment (Figure 3B). This indicated that zinc acetate ameliorated the excess copper-induced impairment of autophagic flux.

Zinc acetate did not change the expression levels of LC3-II and p62 protein (Figure 3C). This showed that zinc acetate alone did not alter autophagic flux.

4-phenylbutyrate (PBA) has been known to act as a chemical chaperone by reducing the load of mutant or misfolded proteins retained in the ER under conditions associated with cystic fibrosis and α1-antitrypsin deficiency [33,34]. Spliced XBP1 and p-eIF2α expressions in cultured hepatocytes were not affected by treatment with PBA alone (Figure 3D). PBA reduced excess copper-induced ER stress, assessed by the expression levels of p-eIF2α and spliced XBP1 (Figure 3E). Moreover, PBA reduced the excess copper-induced elevation of LC3-II and p62 proteins (Figure 3E). In the mRFP–GFP-LC3 transfected cells, PBA significantly reduced the colocalization of mRFP-LC3 with GFP-LC3 induced by excess copper treatment (Figure 3F). PBA did not change the expression levels of LC3-II and p62 protein (Figure 3D). In the mRFP–GFP-LC3 transfected cells, PBA did not affect the colocalization of mRFP-LC3 with GFP-LC3 (Figure 3F). Therefore, PBA alone did not alter autophagic flux. Spliced XBP1, p-eIF2α, and ubiquitinated proteins expressions in cultured hepatocytes were not affected by treatment with zinc acetate alone (Figure 3G). Tunicamycin exacerbated the reduction effects of zinc acetate on increased p-eIF2α, spliced XBP1, ubiquitinated proteins, LC3-II, and p62 expressions by excess copper (Figure 3H). This finding indicated that zinc acetate ameliorated the excess copper-induced impairment of autophagic flux via reducing ER stress.

### 2.4. Zinc Acetate Ameliorates Copper Combined with Proteasome Inhibitor-Induced Mallory–Denk Body-like Inclusion Body Formation

**Figure 4:**
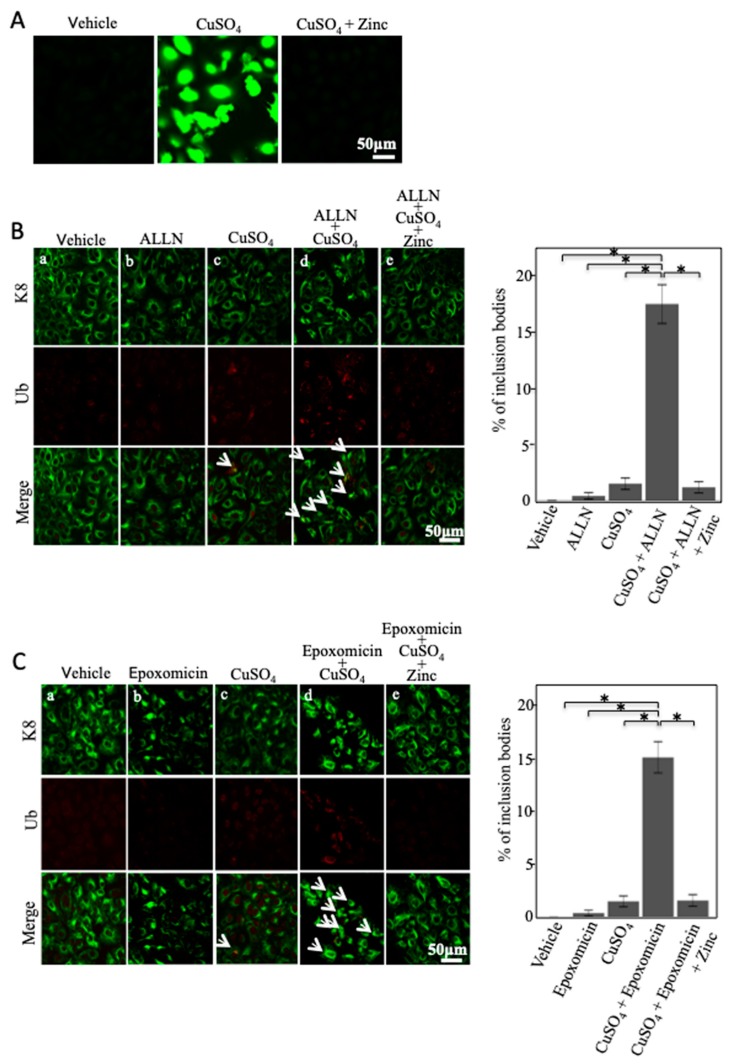
Effect of copper, proteasome inhibitors (PIs), and zinc acetate treatment on Mallory–Denk body (MDB)-like inclusion body formation. (**A**) Cellular hydrogen peroxide assay. OUMS-29 cells were treated with copper at the concentration of 300 μM for 12 h, and zinc acetate (200 μM) was added 2 h before this treatment. That was followed by treatment with 10 μM 2′,7′-dichlorodihydrofluorescein diacetate (H_2_DCFDA) for 30 min, and examined by confocal microscopy. (**B**) OUMS-29 cells were treated with copper at the concentration of 300 μM and acetyl-leucyl-leucyl-norleucinal (ALLN) at the concentration of 10 μM for 12 h. Zinc acetate (200 μM) was added 2 h before these treatments. Cells were then stained by anti-keratin 8 (K8) (green) and anti-ubiquitin (red) antibodies. Arrows indicate the inclusion bodies. For quantitative determination, hepatocytes with inclusion bodies were counted in 20–30 microscopic fields (×250 magnification). Data are expressed as means ± SEM. (**C**) OUMS-29 cells were treated with copper at the concentration of 300 μM and epoxomicin at the concentration of 0.1 μM for 12 h, and zinc acetate (200 μM) was added 2 h before these treatments. Cells were then stained by anti-K8 (green) and anti-ubiquitin (red) antibodies. Arrows indicate the inclusion bodies. For quantitative determination, hepatocytes with inclusion bodies were counted in 20–30 microscopic fields (×250 magnification). Data are expressed as means ± SEM.

We next examined the effect of zinc acetate on oxidative stress in cultured hepatocytes. Oxidative stress was evaluated by the fluorescent signals of 2′,7′-dichlorodihydrofluorescein diacetate (H_2_DFDA). H_2_DFDA signals were increased in cultured hepatocytes treated with excess copper. Zinc acetate reduced these signals (Figure 4A). This indicated that excess copper induced oxidative stress and that zinc acetate reduced this copper-induced oxidative stress.

Moreover, oxidative stress has been reported to contribute to the formation of Mallory–Denk body (MDB)-like inclusion bodies [35]. To evaluate the effect of zinc acetate on MDB-like inclusion body formation, we performed immunofluorescence staining of keratin 8 (K8) and ubiquitin. We defined inclusion bodies as aggregated intermediate filament proteins (K8 and ubiquitin) that have lost their fine intracellular network. We observed excess copper-induced inclusion bodies composed of K8 and ubiquitin by immunofluorescence analysis (Figure 4Bc). However, the effect of copper overload on MDB-like inclusion body formation in cultured hepatocytes was slight (Figure 4Bc). Accordingly, to clarify this effect, we performed experiments in cultured hepatocytes treated with excess copper combined with a small amount of PIs, ALLN, and epoxomicin. We observed a synergistic increase in MDB-like inclusion body formation in cultured hepatocytes co-treated with excess copper and ALLN compared with excess copper only (Figure 4Bd). Zinc acetate dramatically decreased MDB-like inclusion body formation caused by co-treatment of copper and ALLN (Figure 4Be). Epoxomicin also showed similar results to ALLN (Figure 4C). These results indicated that zinc acetate prevented MDB-like inclusion body formation induced by oxidative stress and ER stress.

### 2.5. Zinc Acetate Ameliorates Palmitic Acid-Induced Endoplasmic Reticulum Stress, Impairment of Autophagic Flux, and Apoptosis

We have previously reported that saturated fatty acids (SFAs) impaired autophagic flux at the step of autophagosome–lysosome fusion, and that impairment occurs in an ER stress-dependent manner [21]. Therefore, we examined the effects of zinc acetate on ER stress and autophagic arrest induced by SFAs in cultured hepatocytes.

**Figure 5:**
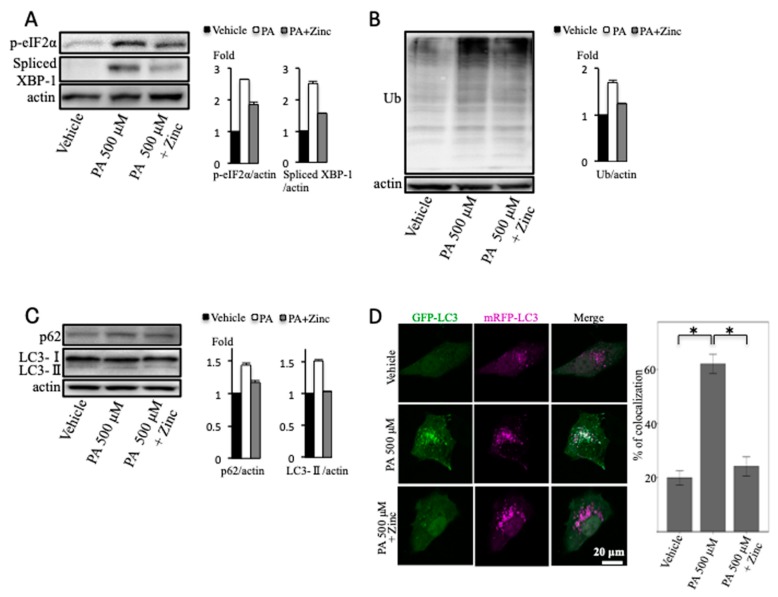
Effect of palmitic acid (PA) and zinc acetate treatment on endoplasmic reticulum (ER) stress and autophagy. (**A**–**C**) Huh7 cells were treated with PA at the concentration of 500 μM for 12 h, and zinc acetate (200 μM) was added 2 h before PA treatment. Densitometry analysis of indicated proteins is shown on the right. (**D**) OUMS-29 cells were transfected with monomeric red fluorescent protein (mRFP)–green fluorescent protein (GFP) tandem fluorescent-tagged light chain 3 (LC3) plasmids. They were treated with PA at the concentration of 500 μM for 12 h, and zinc acetate (200 μM) was added 2 h before PA treatment. Colocalizations of mRFP and GFP signals were measured by counting an overall total of 30 to 40 cells over the course of three independent experiments. Colocalization was shown as the percentage of merged signals in the total number of mRFP signals. Data are expressed as means ± SEM.

We used palmitic acid as SFAs. The elevations of spliced XBP1 and p-eIF2α expression, induced by palmitic acid, were both suppressed in the presence of zinc acetate (Figure 5A). We also found that the level of ubiquitinated proteins increased in the palmitic acid-treated cultured cells and that this effect was ameliorated in the presence of zinc acetate (Figure 5B). With regard to autophagy, zinc acetate reduced the palmitic acid-induced elevation of LC3-II and p62 proteins (Figure 5C). Moreover, in the mRFP–GFP-LC3 transfected cells, zinc acetate significantly reduced the colocalization of mRFP-LC3 with GFP-LC3 induced by palmitic acid treatment (Figure 5D). These results indicated that zinc acetate ameliorated palmitic acid-induced ER stress and impairment of autophagic flux.

## 3. Discussion

Several studies have described the relationship between zinc and ER stress. For example, zinc is necessary for homeostasis of ER functions [36]. Furthermore, it is reported that zinc transporter protein expressions, which maintain intracellular zinc homeostasis, are closely related to the ER stress [37,38,39]. We previously showed that excess copper induced not only oxidative stress but also ER stress in various kinds of cells, including liver-derived cells. DNA damage and apoptosis were also induced by excess copper-induced ER stress. Zinc ameliorated this copper-induced cytotoxicity [12]. In the present study, we demonstrated that zinc acetate ameliorated not only excess copper-induced ER stress but also PIs, including ALLN and epoxomicin-induced ER stress, or tunicamycin-induced ER stress, which operates by inhibiting the first step in the biosynthesis of N-linked glycans, resulting in many misfolded proteins. Thus, we examined the effect of zinc acetate on ER stress induced by various different kinds of stimuli in liver-derived cultured cells, and we showed that zinc acetate ameliorated ER stress in all cases. These findings show that zinc plays a pivotal role in the maintenance of ER homeostasis.

Impairment of protein folding or processing results in the accumulation of misfolded proteins, triggering ER stress, which is a response characterized by ER distension and perturbation in ER homeostasis. To cope with any perturbation by misfolded proteins, the ER induces a cascade of reactions called the UPR [40]. On the other hand, autophagy, which is an intracellular degradation system that delivers cytoplasmic constituents to the lysosome, is a very important reaction for the reduction of ER stress via maintenance of cellular homeostasis, including eliminating abnormal proteins. It is reported that copper induced autophagy and apoptosis via reactive oxygen species (ROS) production and JNK (c-Jun N-terminal kinase) activation in glioma cells [41]. However, few reports are available on the effect of copper on autophagy in hepatocytes. We previously reported that autophagy ameliorated liver injury, and that MDB-like inclusion body formation also protected hepatocytes from oxidative stress [42]. Those results suggested that the induction of autophagy and MDB-like inclusion body formation may be adaptive responses to hepatotoxicity. Furthermore, oxidative stress induced MDB-like inclusion body formation [35]. MDB-like inclusion body formation may be an important reaction to reduce oxidative stress and ER stress-induced cytotoxicity as well as autophagy [42]. Therefore, we examined whether excess copper-induced ER stress induced MDB-like inclusion body formation.

We next examined in detail whether excess copper induced the impairment of autophagic flux. In the present study, LC3 turnover assay and monitoring of mRFP–GFP-LC3 signals indicated that excess copper impaired autophagic flux at the step of autophagosome–lysosome fusion or lysosomal dysfunction. The examination of the localization of Lamp1 and Lysotracker Red indicated normal lysosomal acidification after treatment with excess copper. Furthermore, the examination of Magic Red cathepsin B enzyme activity also showed normal protease activity after treatment with excess copper. We also showed that excess copper impaired autophagic flux in a Rubicon-independent manner. This impairment of autophagic flux showed a pattern similar to the FFA-induced impairment we previously reported [21]. Furthermore, Wilson disease complicates steatohepatitis [43]. We examined the effect of zinc acetate on FFA-induced hepatotoxicity, including the impairment of autophagic flux. Our results showed that zinc acetate ameliorated both FFA-induced hepatotoxicity via the maintenance of ER homeostasis, as well as excess copper-induced hepatotoxicity.

Although excess copper induced the impairment of autophagic flux and the formation of MDB-like inclusion body formation via disruption of ER homeostasis, zinc acetate ameliorated these cytotoxicities via the maintenance of ER homeostasis. Similarly, zinc ameliorated FFA-induced cytotoxicity via the maintenance of ER homeostasis. Our results indicated that zinc ameliorated a variety of cytotoxicities via the maintenance of ER homeostasis. Zinc may be a pivotal agent to protect hepatocytes from ER stress-related diseases.

## 4. Materials and Methods

### 4.1. Cells Cultures and Reagents

We used human hepatoma cell lines established from hepatocellular carcinoma (Huh7) and a highly differentiated immortalized human hepatocyte (OUMS-29) [44]. Huh7 cells were obtained from RIKEN Bioresource center (Tsukuba, Japan). Cells were cultured in Dulbecco’s modified Eagle’s medium (DMEM) supplemented with 10% fetal bovine serum and antibiotics. Cells were maintained in a 37 °C incubator with 5% CO_2_.

The following materials were used: ALLN and epoxomicin as PIs (Calbiochem, La Jolla, CA, USA); copper sulfate (NACALAI TESQUE, Inc., Kyoto, Japan); PBA (Sigma-Aldrich, St. Louis, MO, USA); tunicamycin (Sigma-Aldrich); and zinc acetate (Wako Pure Chemical Industries, Ltd., Osaka, Japan).

Antibodies to the following antigens were used: LC3 (Medical and Biological Laboratories); Beclin 1 (Novus Biologicals, Littleton, CO, USA); Atg7, p-eIF2α (Cell Signaling Technology, Danvers, MA, USA); ubiquitin, XBP1, p62 (Santa Cruz Biotechnology, CA, USA); K8, actin (Sigma-Aldrich); Rubicon (Abcam, Cambridge, MA, USA); and ubiquitin (Dako, Glostrup, Denmark). Lamp1 antibody was a kind gift from J.T. August (Johns Hopkin University, Baltimore, MD, USA).

### 4.2. Immunofluorescence Staining

Cells were fixed in 3% paraformaldehyde (PFA) in phosphate-buffered saline (PBS) for 20 min, and permeabilized with 0.1% Triton X-100 or 0.1% saponin in PBS for 10 min, followed by incubation with the primary antibodies for 1 h at 37 °C. The cells were washed three times with PBS and incubated with the secondary antibodies for 1 h at 37 °C. For double labeling staining, cells were examined using separate excitation wavelengths (488 nm and 543 nm), then merged.

### 4.3. Immunoblotting Analysis

Cells were collected in a lysis buffer composed of 0.187 M trishydroxymethylaminomethane–HCl (pH 6.8), 10% sodium dodecyl sulfate, and 5 mM ethylene diamine tetra-acetic acid, sedimented by spinning and subjected sonication. Equal amounts of protein were separated by polyacrylamide gel electrophoresis (Bio-Rad Laboratories, Hercules, CA, USA). Proteins were transferred to polyvinylidene fluoride microporous membrane (Millipore Corporation, Billerica, MA, USA). After blocking for 1 h at room temperature with 0.2% skim milk in phosphate-buffered saline containing 0.1% Triton X-100, the blot was incubated overnight at 4 °C with the primary antibodies. Membranes were washed three times for 10 min with PBS and incubated with secondary antibodies (horseradish peroxidase-linked sheep anti-mouse antibody and donkey anti-rabbit antibody; GE Healthcare, Buckinghamshire, UK) for 1 h at room temperature. After incubation, the blots were visualized using enhanced chemiluminescence (ECL Plus Western Blotting Detection Reagents; GE Healthcare, Buckinghamshire, UK). The expression of each protein was measured by Light capture (ATTO Corporation, Tokyo, Japan). Results represent the quantification of at least three independent experiments.

### 4.4. Autophagy Analysis

Autophagy was determined by detection of the LC3 protein. Low levels of LC3-II could be due to an inhibition of autophagy or to a high autophagic flux. High levels of LC3-II could be caused by increased autophagosome formation, impairment of autophagosome fusion with lysosomes, or autophagosome degradation. The combinational evaluation of the same sample with or without treatment of bafilomycin A1 (Sigma-Aldrich) allows one to determine whether there has been an induction or inhibition of the autophagic flux.

To analyze autophagic flux, we used mRFP–GFP tandem fluorescent-tagged LC3 (tf-LC3) (Addgene, Cambridge, MA, USA). For the tf-LC3 assay, cells were transfected with plasmids expressing tf-LC3 using Effecten Transfection Reagent (Qiagen GmbH, Hilden, Germany) for 24 h after cell plating. After designated treatments, cells were fixed with 4% PFA in PBS. All the cellular images were obtained using a laser scanning confocal microscope (Zeiss 510 Meta with Lasersharp software, Carl Zeiss MicroImaging Inc., Jena, Germany). Once the autophagosome fuses with the lysosome and matures to an autolysosome, the GFP signal is lost and these structures are recognized in red color only. For assessment of autophagic flux, colocalization of GFP-LC3 and mRFP-LC3 puncta was measured by counting a total of more than 30 cells, and shown as the percentage of the total number of mRFP-LC3 puncta. In addition, to assess late stages of autophagy, cells were transfected with plasmids expressing mRFP-LC3. Colocalization of immunofluorescence staining of Lamp1 and mRFP-LC3 was counted in a total of over 30 cells from each of three independent experiments.

### 4.5. Lysosomal Function Analysis

To analyze lysosomal acidification, cells from different treatments were collected and then incubated with 1 µM Lysotracker Red (Molecular Probes, Eugene, OR, USA) for 1 h at 37 °C, followed by fixation with 4% PFA in PBS. Subsequently, we performed immunofluorescence staining of Lamp1. Lysotracker is a fluorescent dye for labeling acidic organelles, and thus Lysotracker-positive Lamp1 indicates that lysosomal acidification is intact. The catalytic activities of cathepsins were determined by Magic Red Cathepsin B reagent (Immunochemistry Technologies, LLC, Bloomington, MN, USA). Cells were loaded with Magic Red Cathepsin B reagent for 1 h at 37 °C. Red fluorescence can be detected using laser scanning confocal microscopy.

### 4.6. Detection of Reactive Oxygen Species

ROS were detected by analyzing the fluorescence intensity of the intracellular fluoroprobe H_2_DCFDA, a molecular probe for detecting intracellular H_2_O_2_ and oxidative stress [45]. Cells were loaded with H_2_DCFDA at 37 °C for 30 min, and then the cells were examined under a confocal laser scanning microscope.

### 4.7. Transmission Electron Microscopy

A human liver specimen was obtained by liver biopsy for the diagnosis of Wilson disease at the University of Occupational and Environmental Health Hospital (Kitakyusyu, Japan). This examination was approved by the Institutional Review Board of our university (H23–39).

The liver tissue piece was fixed with 2% glutaraldehyde, post-fixed in 1% osmium tetroxide, dehydrated in a graded ethanol series, and embedded in Quetol 812 (Nisshin EM Co., Ltd., Tokyo, Japan). Ultrathin sections were stained with uranyl acetate and lead citrate, and examined by a transmission electron microscope (JEM-1200EX, JEOL Ltd., Tokyo, Japan).

### 4.8. Statistical Analyses

Quantitative data (mean ± SE) were subjected to Kruskal-Wallis test analysis, using IBM SPSS Statistics Version 25 (*p* < 0.05 being significant).

## 5. Conclusions

Our results indicate that excess copper interferes with autophagic flux at the step of autophagosome–lysosome fusion in an ER stress-dependent manner. We demonstrated that zinc acetate restores the impairment of autophagic flux caused by copper overload or lipid overload via reducing ER stress. Zinc acetate also prevents MDB-like inclusion body formation by reducing excess copper-mediated oxidative stress and ER stress, and by reducing autophagic flux impairment. These results suggest that zinc acetate might be therapeutically effective in treating ER stress-related liver diseases.

## Abbreviations

ALLN	acetyl-leucyl-leucyl-norleucinal
Atg	autophagy-related gene
DMEM	modified Eagle’s medium
ER	endoplasmic reticulum
FFA	free fatty acid
GFP	green fluorescent protein
H_2_DFDA	2′,7′-dichlorodihydrofluorescein diacetate
JNK	c-Jun N-terminal kinase
K	keratin
LAMP1	lysosomal-associated membrane protein 1
LC3	light chain 3
MDB	Mallory–Denk body
mRFP	monomeric red fluorescent protein
PBA	4-phenylbutyrate
PBS	phosphate buffered saline
p-eIF2α	phospho-α-subunit of eukaryotic initiation factor 2
PFA	paraformaldehyde
PI	proteasome inhibitor
PI3K	phosphatidylinositol-3 kinase
ROS	reactive oxygen species
Rubicon	Run domain Beclin1-interacting and cysteine-rich containing protein
SEM	standard error of mean
SFA	saturated fatty acid
tf-LC3	tandem fluorescent-tagged LC3
UPR	unfolded protein response
XBP1	X-box binding protein 1
